# Use of Basket Trials to Solve Sleep Problems in Patients with Rare Diseases

**DOI:** 10.3390/clockssleep6040044

**Published:** 2024-11-05

**Authors:** Lara C. Pullen, Nick Bott, Cate McCanless, Amee Revana, Gunes Sevinc, Casey Gorman, Alexandra Duncan, Sarah Poliquin, Anna C. Pfalzer, Katie Q. Schmidt, E. Robert Wassman, Chère Chapman, Maria Picone

**Affiliations:** 1 Chion Foundation, Oak Park, IL 60304, USA; 2 Takeda Pharmaceuticals, Cambridge, MA 02139, USA; nick.bott@takeda.com; 3 Harmony Biosciences, Plymouth Meeting, PA 19462, USA; cmccanless@harmonybiosciences.com; 4 Texas Children’s Hospital, Houston, TX 77001, USA; axpatel1@texaschildrens.org; 5 Ardea Outcomes, Halifax, NS B3J 0J2, Canada; gunes.sevinc@ardeaoutcomes.com (G.S.); chere.chapman@ardeaoutcomes.com (C.C.); 6 Department of Neurology, Perelman School of Medicine, University of Pennsylvania, Philadelphia, PA 19104, USA; casey.gorman@pennmedicine.upenn.edu; 7 COMBINEDBrain, Brentwood, TN 37027, USA; alexandra.duncan@combinedbrain.org (A.D.); sarah@combinedbrain.org (S.P.); anna.c.pfalzer@vumc.org (A.C.P.); katie.schmidt@combinedbrain.org (K.Q.S.); 8 Vanderbilt University Medical Center, Nashville, TN 37232, USA; 9 TREND Community, Philadelphia, PA 19102, USA; drbobwassman@gmail.com (E.R.W.); maria@trend.community (M.P.)

**Keywords:** clinical trials, drug evaluation, patient care, rare diseases, sleep disorders

## Abstract

The need for sleep is universal, and the ability to meet this need impacts the quality of life for patients, families, and caregivers. Although substantial progress has been made in treating rare diseases, many patients have unmet medical sleep needs, and current regulatory policy makes it prohibitively difficult to address those needs medically. This opinion reviews the rare disease experience with sleep disorders and explores potential solutions. First, we provide case profiles for the rare diseases Wilson’s Disease, Angelman Syndrome, and Prader–Willi Syndrome. These profiles highlight challenges in rare disease diagnosis and barriers to pinpointing disease pathophysiology, including biomarkers that intersect with sleep disorders. Second, we transition to a bird’s eye view of sleep disorders and rare diseases by reporting input from a stakeholder discussion with the U.S. Food and Drug Administration regarding abnormal sleep patterns in various rare diseases. Last, in response to the profound unmet medical needs of patients with rare diseases and sleep disorders, we propose adapting and using the clinical trial design known as a “basket trial”. In this case, a basket trial would include patients with different rare diseases but the same debilitating symptoms. This research approach has the potential to benefit many rare disease patients who are otherwise left with profound unmet medical needs.

## 1. Introduction

The need for sleep is universal, and the ability to meet this need drives the quality of life for individuals, families, and caregivers [1,2]. According to the National Institute of Neurological Disorders and Stroke, sleep is required for the proper upkeep of almost all body systems and tissues, including brain function. It is “as essential to survival as food and water” [3]. Abnormal sleep may cause problems with metabolism, decision-making, cognitive ability, mood, and increased vulnerability to seizures [4,5,6].

## 2. Unmet Sleep Needs in Patients with Rare Diseases

The American Academy of Sleep Medicine and Sleep Research Society agree that sleep is essential for optimal health and specify that “Healthy sleep requires adequate duration, good quality, appropriate timing and regularity, and the absence of sleep disturbances or disorders” [7]. Many patients with rare diseases have unmet sleep needs [8,9,10,11,12,13]. Although each rare disease affects fewer than 200,000 individuals in the United States, there are approximately 7000 rare diseases [14]. This means that an estimated 25–30 million Americans have a rare disease, and many more provide care for an individual with a rare disease [15]. While the Orphan Drug Act has facilitated the approval of drugs for rare diseases, multiple challenges remain in bringing these drugs to market. For example, intrinsic limitations in the patient pool make it difficult to enroll patients at the necessary level to attain statistical significance. Additionally, clinical trials must de facto compete against other trials when enrolling patients [16]. In the case of sleep disorders, a lack of biomarkers of sleep and insomnia presents an additional challenge to clinical trial design [17].

The sleep problems experienced by patients with rare diseases include disrupted circadian rhythms [18,19,20], possibly due to an inability to synchronize the sleep–wake cycle with the light–dark cycle [9], leading to conditions such as insomnia or excessive daytime sleepiness (EDS), also known as hypersomnolence [10]. Unfortunately, rare diseases are often poorly understood, and their many manifestations, including sleep disorders, can have complex etiologies [16]. In this opinion article, we review the experience of rare diseases in relation to sleep disorders and propose a clinical trial approach with the potential to benefit many patients with rare diseases who are otherwise left with profound, unmet medical needs.

### 2.1. The Patient Odyssey: Wilson’s Disease

Although many clinicians do not consider sleep disorders to be fundamental to Wilson’s Disease (WD), a rare disease with a prevalence of 1 in 30,000 to 50,000 [21], research has documented insomnia, daytime sleepiness, cataplexy-like episodes, and sleep paralysis as common and often underdiagnosed symptoms of this condition [22]. A recent case report described a 21-year-old patient with WD whose initial manifestation was acute insomnia. His difficulty falling asleep manifested suddenly and was associated with fatigue and memory problems, as well as a feeling of distress. After two months of acute symptoms, the patient was diagnosed with WD and received anti-insomnia treatments, as well as standard WD treatment. Despite this, he experienced only slight improvements in his neurologic and insomnia symptoms [23].

As with many rare diseases, clinicians lack detailed data on the presentation of sleep disturbances in patients with WD. Early manifestations vary substantially; while WD patients classically present with liver disease, many have neurological or psychiatric symptoms at onset, including dystonia, ataxia, and sleep disorders [24]. Absent a biomarker for sleep disorders, patients often struggle to achieve an accurate clinical diagnosis. In this situation, sleep disorders are not likely to be considered or treated as primary symptoms [25,26,27]. Nonetheless, a meta-analysis found that 54% of patients with WD experience sleep disorders [28], and direct comparisons of WD patients to healthy controls confirm that patients have significantly worse sleep quality, less sleep efficiency, increased wakefulness after sleep onset, and more arousal [28,29]. These sleep disorders have been shown to persist even with standard therapeutic interventions [29].

Experts suggest that the high prevalence of sleep disorders in WD could be related to the pathologic accumulation of copper in the sleep–wake pathways in the brain, WD treatment, and/or other WD-related symptoms [23,30]. It has been noted that brain damage accrual in WD is more likely with delayed diagnosis and treatment [31]. Unfortunately, while early diagnosis and treatment may slow the cascade of brain tissue destruction associated with copper accumulation in the brain, the rare disease diagnostic odyssey is typically long [31,32].

### 2.2. Challenges in Determining Pathophysiology: Angelman Syndrome

Angelman Syndrome (AS) is a rare and severe neurodevelopmental disorder, with a prevalence estimated at 1 in 12,000 to 20,000 [33]. Widespread sleep disturbances in patients with AS have been confirmed by both surveys of parents and caregivers and polysomnographic (PSG) studies based on electroencephalogram (EEG) data [9]. Individuals with AS lack *ubiquitin protein E3A ligase gene* (*UBE3A*) expression in the brain, and mutations in the *UBE3A* allele appear to underly all AS disabilities [9,34].

Based on expert consensus, the AS diagnostic criteria include sleep disturbances, abnormal sleep–wake cycles, and a diminished need for sleep [35]. Patients have widespread electroencephalogram (EEG) disturbances, with two of the most commonly reported abnormalities being intervals of amplitude slow waves and interictal epileptiform spike-wave discharges [36]. EEG studies also indicate decreased sleep efficiency and differences in the number and duration of non-rapid eye movement (NREM) sleep spindles [37].

Although sleep research in mice is challenging, investigators have used EEG and behavioral measures of sleep pressure to study changes in the homeostatic mechanism underlying sleep disorders in AS [9]. Research using a maternal *Ube3a* deletion mouse model found pronounced deficits in NREM delta power and subsequent NREM sleep patterns [38]. In this study, AS mice forcibly deprived of sleep during early daytime (when mice are sleeping most intensely) displayed blunted changes in wave-incidence and NREM delta power accumulation and less recovery sleep during sleep deprivation. These findings suggest that UBE3A loss reduces sleep pressure. However, research is ongoing to establish a cause–effect relationship between circadian dysfunction and sleep disorders in patients with AS and to clarify the potential interactions between UBE3A and core circadian clock proteins [9].

Some AS investigators have proposed that patient sleep disturbances reflect reduced sleep efficiency. In a mouse study, AS model mice experienced more transitions between sleep states and decreased depth of NREM sleep and showed a trend for decreased REM amount during undisturbed sleep compared to wild-type mice [39]. These traits mirror symptoms seen in patients with AS [40]. The generalization from mice to humans remains challenging, however, for species-level differences in nocturnality and diurnality [9].

Studies comparing melatonin secretion patterns in children with AS to healthy children have found that children with AS had significantly lower nighttime melatonin levels, even though the total sleep time and the ratio of nocturnal to total sleep time did not differ between the two groups [41]. In children with AS, individual melatonin secretion curves are highly variable [9,42], and the duration of nighttime melatonin secretion is prolongued [9]. Unfortunately, the shortage of studies, small sample sizes, high individual variability, and differences in patient characteristics make it difficult to draw conclusions. Research is limited and inconsistent, and the available studies are small, but data suggest that melatonin administration improves sleep latency, efficiency, duration, and nighttime awakenings for most patients with AS [42,43,44]. Nonetheless, sleep disturbances persist in other patients, suggesting an underlying pathophysiology that is more complex than mere melatonin deficiency [42,43]. Mouse models have also yielded conflicting results, possibly because different background mouse strains produce different melatonin levels [38,45]. Thus, despite numerous patient and animal studies, the AS community lacks a consistent standard for research on sleep in individuals with AS, and experts remain uncertain about the underlying cause of sleep problems in AS [13].

### 2.3. Finding Potential Solutions: Prader-Willi Syndrome

While many patients with rare diseases experience sleep disorders, only a select few rare diseases benefit from being studied for the development of drugs targeting sleep. The Prader–Willi Syndrome (PWS) is one such disease, with a prevalence between 1 in 10,000 to 30,000 individuals [46].

While the hallmark symptom of PWS is hyperphagia, sleep disturbances are common [10]. Similar to other rare diseases, PWS is characterized by a lack of established biomarkers, which has served as a barrier to conducting clinical trials [16]. Only one medication (recombinant human growth hormone, approved in 2000) is currently approved for PWS [16,47]. The combination of growth hormone treatment and non-pharmacological interventions has improved the stature, body composition, and overall quality of life for this patient population [48]. Unfortunately, even with this treatment, EDS, hyperphagia, and behavioral dysregulation remain severe and, at times, life-threatening for patients [16].

Both real-world and phase 2 proof-of-concept research indicated that a medication (pitolisant) relieved excessive daytime sleepiness, normalized nighttime sleep, and improved cognitive function in this patient population [10,49,50]. Initially approved in 2019, the FDA currently indicates pitolisant for the treatment of EDS or cataplexy in adult patients with narcolepsy [51], and the sponsor is currently pursuing a second indication for PWS [52].

## 3. The Need for Better Pathways

WD, AS, and PWS serve as three examples of the journey that rare disease communities experience as they seek solutions for the sleep disorders that undercut the quality of life. Solutions to the ubiquitous sleep problems in the rare disease community should have been made more accessible by the US Congress’ passage in 1983 of the Orphan Drug Act [53,54], which comprised a collection of push and pull policies designed to increase the number of drugs developed for rare diseases [55]. Many pharmaceutical companies took advantage of the Orphan Drug Act, and between 1983 and 2022, the FDA granted orphan designation to 6340 drugs. Of these, 882 resulted in FDA approvals across 392 rare diseases. Despite this success, orphan drugs are being developed for only 15% of rare diseases, and FDA-approved treatments exist for only 5% [56].

Some incentives exist for manufacturers. Special FDA designations have the advantage of shortening clinical development and FDA approval times for new drugs that treat rare diseases with unmet medical needs [55]. The FDA also requires a shorter review period for orphan drugs when a sponsor applies to expand a label to another rare disease. However, the current single-disease approach to clinical trials makes obtaining FDA indications for more than one or two rare diseases almost impossible before a drug’s patent expires. This is because, while extension possibilities exist, a typical patent lasts 20 years, and preclinical and clinical research can last 5–10 years [57]. In most cases, therefore, it would be difficult, if not impossible, for a sponsor to financially justify pursuing more than one or two additional rare disease indications before generic competitors enter the marketplace.

While the passage of the Orphan Drug Act has benefited the rare disease community, it has also been associated with unintended financial consequences. Owing to small patient populations, rare disease drugs typically carry a higher price tag to deliver a return on investment. Pressure on payers is multiplied when these high individual drug costs are considered in context with the increasing numbers of rare disease drugs approved [58,59]. Between 1992 and 2019, the proportion of overall US drug spending dedicated to orphan drugs rose from 2% to 11% [59,60]; concurrently, a 2017–2021 analysis of newly approved US medications found that orphan drugs had a median treatment cost 17 times higher than non-orphan drugs [61]. In other cases, patients with rare diseases who might benefit from a given drug will be limited to off-label use, which is also associated with extreme access barriers. Patients often have trouble obtaining insurance coverage for off-label use of these expensive medications [62,63], and direct drug purchases may not be financially feasible.

Thankfully, the FDA has regulatory flexibility in approving drugs and has a history of using this flexibility to approve drugs to treat neurodegenerative disorders such as Alzheimer’s disease and amyotrophic lateral sclerosis [16]. The FDA has also applied this flexibility to the orphan drug approval process, encouraging sponsors to use continuous development trial designs that allow rare disease patients to transition smoothly from one trial to another, at times dismissing the requirement of a complete phase 2 and phase 3 study, and encouraging the use of a natural history data as a control. Unfortunately, the FDA continues to emphasize the importance of sufficiently well-described mechanisms of action for agents and the importance of biomarkers [16,64,65]. As detailed in the examples of WD and AS, such a reliance on understanding pathophysiology and using biomarkers presents an undue burden for rare disease communities.

For example, Wilson’s Disease Association does not mention sleep but discusses liver, neurologic, and psychiatric symptoms [66]. The Angelman Syndrome Foundation lists clinical trials targeting increased production of UBE3A protein, improving the impaired connections and signaling between brain cells, and restoring the GABA function [67]. None of the studies specify improved sleep as an outcome. Instead, they focus on what the community considers to be the core features of the disease.

Should a sleep-specific clinical trial attempt to enroll AS patients, the trial would compete against the five other trials currently listed on the Angelman Syndrome Foundation website [67]. Such competition would increase the difficulty of fully enrolling all the trials. Moreover, given resource scarcity, few communities can retreat from core problems to perform the research necessary to identify the pathophysiology of the sleep issues experienced by individuals with a given rare disease. By neglecting sleep, however, we are pushing aside the disproportionate effect of sleep disturbance on a patient’s quality of life [68,69]. This results in a constellation of patient, caregiver, and clinician communities that lack solutions for debilitating symptoms. In recognition of this, many in the rare disease community have called on the FDA to apply the broadest regulatory flexibility to the statutory standards, embrace creative strategies, and use clinically grounded scientific judgment to facilitate the approval of rare disease drugs [27,64,70,71].

## 4. Rare Disease Sleep Summit and Critical Path Innovation Meeting

**Figure 1:**
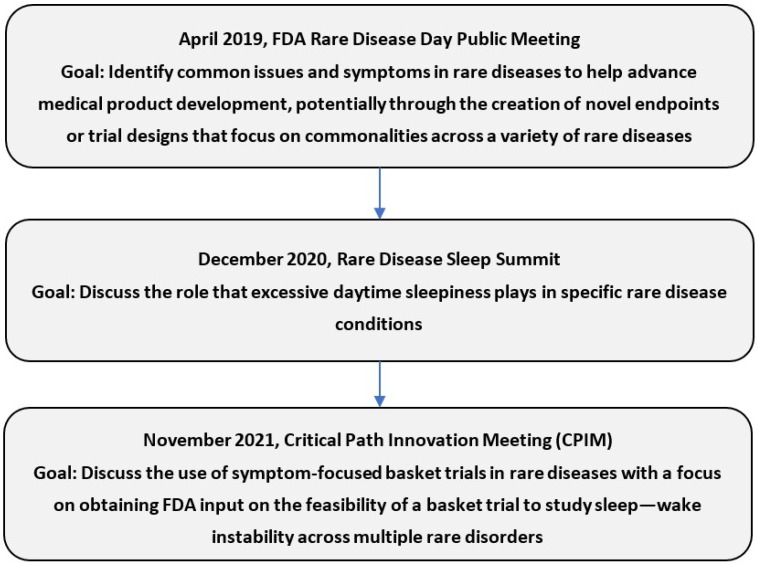
Timeline of stakeholder engagement.

The rare disease community has spent several years gathering data about the importance of sleep in rare diseases and formulating solutions to the many sleep problems affecting the community. In April 2019, the FDA catalyzed this process by engaging with rare disease communities in a public meeting, “Patient Perspectives on the Impact of Rare Diseases: Bridging the Commonalities” [72]. This meeting prompted a Rare Disease Sleep Summit (the Summit), held virtually in December 2020 (Figure 1) [27].

The Summit consisted of advocacy leaders, parents, and caregivers representing rare, neurological disorders where patients commonly report sleep disturbances. While some patients were included in the process, in many cases, patients were non-verbal and could not answer those questions for themselves. Stakeholders included TREND Community (a digital health analytics company founded by parents of a child with PWS), COMBINEDBrain (a non-profit consortium of 100 patient advocacy organizations representing rare genetic neurodevelopmental disorders), the Sleep Consortium (a non-profit dedicated to accelerating global research and therapeutics in central disorders of hypersomnolence), Ardea Outcomes (a contract research organization pioneering patient-centric outcome measurement techniques), and various sub-specialists, including a pediatric sleep specialist from the Texas Children’s Hospital Sleep Center [27].

During the Summit, the moderator asked 12 open-ended questions (Table 1) focused on three areas: (1) physical, emotional, social, and cognitive challenges of EDS and the impact of these challenges on daily functioning; (2) the challenges associated with disorders of sleep–wake stability in patients with other primary symptoms; and (3) the overall impact of sleep–wake instability and related symptoms on the burden of illness, caregiver and family strain, and finances [27].

The summit generated real-world, first-hand insight into the impact of EDS on the patients’ quality of life and functioning (Table 2). While it is impossible to generalize from the responses from these stakeholders to all patients of all rare diseases, the answers nevertheless shed light on a rarely queried topic. The overwhelming input was that abnormal sleep, especially EDS, is a significant and inadequately treated health problem. Participants reported that sleep disruption impaired physical health/fatigue, activities of daily living, socialization/emotional stability, cognition, and finances. Findings also indicated that EDS and subsequent impaired daytime functioning in patients with rare diseases are underrecognized by healthcare professionals, caregivers, and, for some diseases, even by the patients themselves [27].

Summit participants also described how sleep-related conditions undermined their social interactions, ability to build and maintain a support network, and academic and career development. They explained that cognitive impairment resulting from sleep instability ranged from “brain fog” to severely decreased memory and attention. Participants also shared high financial costs and difficulties experienced when searching for physicians who could adequately address their sleep needs. Many stakeholders described problems obtaining recognition from healthcare providers that sleep plays a critical role in quality of life. Lastly, participants shared information about the tremendous burden of sleep disorders on caregivers [27].

Summit stakeholders sought a creative strategy to obtain regulatory solutions to achieve pharmacologic treatment for shared, debilitating sleep symptoms. They concluded by proposing an adaptation of oncological “basket trials” to rare diseases, using a trial design that includes patients with different rare diseases but the same debilitating symptoms [27]. The National Cancer Institute defines a basket trial as a clinical trial that tests how well a new drug or other substance works in patients with different types of cancer with the same mutation or biomarker. In basket trials, patients all receive the same treatment, which targets the specific mutation or biomarker found in their cancer [73,74]. This trial design originated in oncology to test a targeted therapy on a broad spectrum of cancers with a shared biomarker [75]. While investigators have discussed the use of basket trials for other conditions, such as neurogenerative disorders, their use has not yet expanded beyond oncology [76].

**Figure 2:**
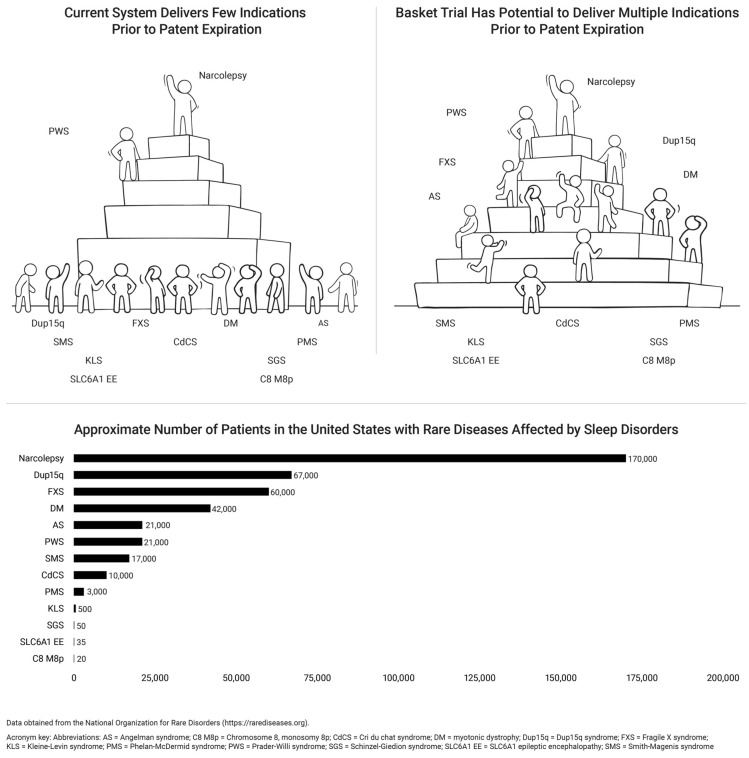
Basket trial design benefits rare disease patients.

The existence of such a path to drug approval could provide sponsors with the information they need to selectively pursue simultaneous indications for EDS in multiple rare diseases, thereby allowing the treatment of more patients than is feasible under the current drug approval system (Figure 2). In November 2021, stakeholders brought this idea to a Critical Path Innovation Meeting (CPIM) with the FDA to seek FDA’s input on the feasibility of a symptom-focused basket trial to study EDS across multiple rare disorders. (Figure 2) [64].

## 5. FDA CPIM Response

FDA representatives explained that, at a high level, the FDA is open to treating common symptoms across multiple diseases. As a prerequisite to evaluating pooled disease states in a basket trial, however, sponsors must provide a compelling mechanistic rationale that includes detailed symptom summaries and an explanation of commonalities in how symptoms manifest across diseases. In the case of EDS, the FDA would need a clear definition of the disease state, identification criteria, and documentation that the condition has a similar presentation across the rare diseases proposed for evaluation [64].

The FDA explained that a basket trial would only be justified if there were a strong pathophysiology reason to hypothesize that the drug could benefit all diseases in the trial. Moreover, assessments and outcome measures must be consistent across all diseases evaluated. The FDA also underscored the difficulty in identifying placebo control data for a basket trial design with multiple disease groups [64].

The meeting ended with the FDA noting that, once pre-clinical data were gathered across potential indications, the sponsor should discuss the trial design with the relevant FDA division (psychiatry in the case of EDS). For the pre-investigational new drug (IND) meeting, the division would require details about the symptoms, population, and drug, and would provide high-level feedback on clinical trial design. If the proposal were to repurpose a drug, the sponsor would also be required to provide details on chemistry, manufacturing, and other practical matters necessary for an IND submission [64].

## 6. Our Response

We feel that the FDA response ignores the significant burden which providing a compelling mechanistic rationale would place on each rare disease. Moreover, the complexity of each rare disease, the spectrum of presentation, and its evolution over a lifetime make it incredibly difficult to document that EDS has a similar presentation across even one rare disease, let alone multiple rare diseases. We thus continue to urge the FDA to execute its regulatory flexibility to embrace creative strategies based on clinically grounded scientific judgment. Even unsuccessful study results could yield insights into potential differences in the underlying pathophysiology between rare disease states, revealing new directions for research and thereby creating further opportunities for solutions.

We propose that the FDA create a basket trial pathway to allow for the label expansion of approved drugs to include sleep disturbances across multiple rare diseases. We acknowledge that sleep disturbances have multidimensional and complex etiologies and that, for most rare diseases, the pathophysiology of sleep is poorly understood or characterized. We recognize it is unlikely that all rare diseases would benefit from the same medication. Patients with rare diseases who enrolled in such a trial would thus be informed they are participating in a clinical study for which the mechanism of action is uncertain. Patient history, subjective questionnaires, evaluation by a sleep medicine specialist, and a sleep study with multiple sleep latency testing may be used to define EDS [77,78], and personalized endpoints can be used to assess treatment outcomes [79]. We propose that patients be randomized to active treatment or control, with a trial extension for active treatment. Such a development path could replace single rare disease “winners” with a “victory team” of rare diseases, all of which could achieve FDA approval for on-label use of an effective drug (Figure 2). Industry stakeholders, including the authors of this article, have indicated that if such a path were available, they would partner with rare disease patients to work towards more equitable access to sleep.

## Figures and Tables

**Table 1:** Open-ended questions discussed during the summit on sleep disruption.

How does EDS impact physical health? If you think about the day-to-day life of sleep disruption, how would you describe to someone how this affects your life day to day? When you think about being a parent of a child with sleep issues, how do you separate behavioral issues that are normal from things that are based on sleepiness? How is the workforce and advancement impacted? Do people not have control of their emotions? Is this a real problem? Does it make it easier because you go through long periods of normality or harder because out of the blue your life is disrupted? How does that impact relationship building? What is the financial impact of EDS? What kinds of resources and support are available to you to deal with EDS? What is missing? In your experience in talking to doctors about sleep issues, how has that gone? How common is it in a sleep issue to have a multidisciplinary team to coordinate issues? How do patients choose what treatments to use?

EDS = excessive daytime sleepiness. Adapted from the Rare Disease Sleep Summit White Paper: Picone, M.; Naujokas, M.F.; Gorman, C.; Jesteadt, L.; Kelly, E.; Kelly, J.; Bichell, T.J. Underrecognized Sleep Disorders Across Rare Diseases: Real-World Insights from a Patient and Caregiver Summit. TREND Community 9 June 2021. Available online: https://doi.org/10.21203/rs.3.rs-555846/v1 (accessed on 2 November 2023) [27].

**Table 2:** Burdens reported by summit participants related to sleep disruption.

Category	Burdens
Physical health/fatigue	Weight loss, muscle loss, pain from lying in bed for many hours, poor nutrition, high blood pressure, frequent fatigue, reduced physical endurance, reduced strength, falling asleep during the day
Activities of daily living	Dependent upon others for housecleaning and cooking, missed school, little time for homework, poor quality homework, lost jobs
Socialization/emotional	Anxiety, depression, emotional outbursts, behavioral outbursts, reduced emotional regulation, stress from poor school performance, social isolation, missed social opportunities, isolation from depression, moods that stress relationships, limits to caregiver social life, avoiding dating
Cognition/neurological	Foggy brain, reduced attention span, memory loss, slowed processing speed, loss of consciousness, drop attacks interpreted as tantrums, poor concentration during work and school
Financial	Reduced or no income, reduced ability to work, costs to pay others to do daily tasks such as housework and cooking, costs associated with reduced independence and reliance on care, costs of travel to disease specialists, costs of finding specialized doctors, high credit card bills to pay for medications

Adapted from the Rare Disease Sleep Summit White Paper: Picone, M.; Naujokas, M.F.; Gorman, C.; Jesteadt, L.; Kelly, E.; Kelly, J.; Bichell, T.J. Underrecognized Sleep Disorders Across Rare Diseases: Real-World Insights from a Patient and Caregiver Summit. TREND Community 9 June 2021. Available online: https://doi.org/10.21203/rs.3.rs-555846/v1 (accessed on 2 November 2023) [27].
